# Effects of Plant Hormones, Metal Ions, Salinity, Sugar, and Chemicals Pollution on Glucosinolate Biosynthesis in Cruciferous Plant

**DOI:** 10.3389/fpls.2022.856442

**Published:** 2022-04-28

**Authors:** Zeci Liu, Huiping Wang, Jian Lv, Shilei Luo, Linli Hu, Jie Wang, Lushan Li, Guobin Zhang, Jianming Xie, Jihua Yu

**Affiliations:** College of Horticulture, Gansu Agricultural University, Lanzhou, China

**Keywords:** glucosinolate, cruciferous plant, secondary metabolite, hormone, exogenous substance

## Abstract

Cruciferous vegetable crops are grown widely around the world, which supply a multitude of health-related micronutrients, phytochemicals, and antioxidant compounds. Glucosinolates (GSLs) are specialized metabolites found widely in cruciferous vegetables, which are not only related to flavor formation but also have anti-cancer, disease-resistance, and insect-resistance properties. The content and components of GSLs in the Cruciferae are not only related to genotypes and environmental factors but also are influenced by hormones, plant growth regulators, and mineral elements. This review discusses the effects of different exogenous substances on the GSL content and composition, and analyzes the molecular mechanism by which these substances regulate the biosynthesis of GSLs. Based on the current research status, future research directions are also proposed.

## Introduction

As one of the most important leafy vegetables, cruciferous plants are cultivated and consumed throughout the world. Most cruciferous vegetables can be eaten fresh or cooked; meanwhile, the leaves can also be used as animal feed, and the seeds can be used to produce edible oil. Cruciferae supply a multitude of health-related micronutrients and phytochemicals (Nilsson et al., 2006; Hanschen and Rohn, 2021). With the pursuit of healthy lifestyles, the consumption of the Cruciferae is increasing, and the planting area is expanding, according to the statistics of the Food and Agriculture Organization of the United Nations (FAOSTA, 2019). Studies have shown that the Cruciferae are enriched for antioxidant compounds, especially glucosinolate.

Glucosinolate (GSL) is a type of anion hydrophilic specialized metabolite containing nitrogen and sulfur; over 15 types of GSLs were detected in the Cruciferae, and the GSL contents are relatively high in the Cruciferae compared with other species (Hwang et al., 2019). The GSL biosynthesis pathway is complex and mainly consists of three stages: side chain extension, core structure formation, and secondary modification. The side chain extension includes deamination, condensation, isomerization, and oxidative decarboxylation, involving *BCAT*, *MAM*, and other gene families. Biosynthesis of core structures consists of five biochemical steps, including oxidation, conjugated oxidation, C-S cleavage, glycosylation, and sulfidation, mainly involving *CYP79* and *CYP83* gene families. Side chain modifications lead to different types of GSLs; *FMOGS-OX* and *AOP* gene families are involved in side chain modification of aliphatic GSL; *CYP81F* and *IGMT* gene families are involved in indole GSL, respectively (Harus et al., 2020). Studies have shown that *R2R3-MYB* transcription factors play important roles in regulating the biosynthesis of aliphatic and indole GSL (Sonderby et al., 2010a; Kumari et al., 2019). *MYB28*, *MYB29*, *MYB34*, *MYB51*, *MYB76*, and *MYB122* participate in the regulation of GSL biosynthesis, among which *MYB28*, *MYB29*, and *MYB76* regulate the biosynthesis of aliphatic GSL (Gigolashvili et al., 2008; Cai et al., 2018). *MYB34*, *MYB51*, and *MYB122* regulate the biosynthesis of indole GSL (Frerigmann et al., 2014; Mitreiter and Gigolashvili, 2021). Naturally occurring GSL have a common chemical structure: the structures are generally composed of β-D-glucosaminyl, a sulfide oxime group, and side-chain R groups derived from amino acids; furthermore, GSL exist generally in the form of potassium or sodium salts (Fahey et al., 2001). Based on the amino-acid side chain R groups, GSLs can be divided into three categories, including aliphatic GSL (side chains are mainly derived from methionine, alanine, valine, leucine, or isopropyl leucine), indole GSL (side chain derived from tryptophan), and aromatic GSL (side chains derived from phenylalanine or tyrosine) (Sonderby et al., 2010b; Bekaert et al., 2012).

The GSL are generally stable in the cytoplasm of cells, while the myrosinase that hydrolyzes these compounds is located in vacuoles (Bhat and Vyas, 2019). When plant tissue is damaged, GSL produce a large number of different hydrolysates under the catalysis action of myrosinase. The initial enzymolysis products are unstable glycoside ligands and D-glucose, and the glycoside ligands are hydrolyzed or rearranged to produce different products. Under acidic conditions, the products are mainly nitriles, while these products mainly form thiocyanates through loosened rearrangement under neutral conditions. In the presence of the EPS (extracellular polymer) enzyme, Fe^2+^ and with unsaturated bonds at the end of the R group, the degradation products were mainly cyclic thionitrile. When R is indole or benzene and its derivatives, thiocyanates will be rearranged, and the degradation products spontaneously cyclize to form azolidinone when R contains the hydroxyl group. The varieties of the GSLs hydrolysis products lead to a wide range of biological activity. GSL are not only related to the flavor of cruciferous vegetables (Anthony et al., 2020; Connolly et al., 2021; Shin et al., 2021) but also play important roles in protecting plants from insect and microbial pathogens (Kunierczyk et al., 2008; Robert et al., 2019; Yao et al., 2019). Some GSL, such as glucoraphanin, play a significant role in the prevention of certain chronic diseases, including hypertension, diabetes, neurodegenerative diseases (Parkinson’s and Alzheimer’s diseases), some types of cancers, and certain cardiovascular diseases (Folmer et al., 2014; Lee et al., 2019). However, the degradation of 2-hydroxyl-3-butenyl GSL adversely affects animal growth, reproduction, and also causes goiter and abnormalities in internal organs of animals (Angelino and Jeffery, 2021). Because the GSL have great influence on plants, animals, and humans, the GSL biosynthesis and functional studies are one of the hot topics in international research at present. Medical specialists focus on the therapeutic effects of GSL, while the breeders are more concerned with reducing harmful GSL and increasing GSL content, which are beneficial to humans and have pest-control effects. Therefore, studying the change mechanism of GSL content and components induced by different factors is of great significance to increase the quality of cruciferous vegetables, enhance the resistance to disease and insect pests, and extract beneficial GSL components.

Studies have shown that the main factor affecting the content and composition of GSL is genotype, and the content and composition of GSL were different among different species and varieties (SzydłOwska-Czerniak et al., 2011; Wiesner et al., 2013b; Rhee et al., 2020; Jeon et al., 2022). Temperature, light quality, mechanical injury, and insect feeding can affect GSL biosynthesis and component content (Pedreno et al., 2017; Kim D. et al., 2018; Lin et al., 2022). Moreover, biosynthesis of GSL is also influenced by plant hormones, plant growth regulators, chemical fertilizers, pesticides, metal ions, gas, etc. (Barickman et al., 2013; Bakhtiari et al., 2018; Li et al., 2018; Mao et al., 2018; Teng et al., 2021). Although there are many reports about the effects of these chemicals on GSL biosynthesis, there is no review of the effects of these substances. This review explores and summarizes the effects of plant hormones, plant growth regulators, mineral elements, heavy metals, antibiotics, and other chemicals substances on GSLs biosynthesis. The present review could provide a theoretical basis for the application of GSLs in regulating the content of certain GSLs and high-quality production of cruciferous vegetables.

## Plant Growth-Regulating Substances

### Jasmonic Acid and Methyl Jasmonate

As an endogenous growth regulator in higher plants, jasmonic acid (JA) can inhibit plant germination, growth, and improve plant resistance (Pangesti et al., 2016; Zhou and Johan, 2017; Mao et al., 2018). In the regulation of GSL biosynthesis, Traw et al. (2003) found that JA increased the total GSLs in *Arabidopsis thaliana* (Traw et al., 2003). After JA treatment, 1-methoxy-indol-3-ylmethyl in the culture medium cultivated Pak Choi (*Brassica rapa* L.) hairy roots and the glucobrassicin, indole-3-carbinol, 1-methoxy-indole-3-carbinol, and total GSLs contents in the broccoli (*Brassica oleracea* L. var. *italica*) suspension-cultured cells increased (Kastell et al., 2013; Wiesner et al., 2013a). Besides, the JA increased sinigrin, gluconapin, progoitrin, glucoiberin, and total GSLs concentrations consistently in cabbage (*B. oleracea* L. var. *capitata* L.) (Bruinsma et al., 2007; Fritz et al., 2010). Accumulation of 4-hydroxyglucobrassicin, 4-methoxyglucobrassicin, glucobrassicin, neoglucobrassicin, and total aliphatic GSLs increased significantly after application of JA in turnip (*B. rapa* L. subsp. *rapa*) and broccoli (*B. oleracea* L. var. *italica*) sprouts (Thiruvengadam et al., 2016b; Guo et al., 2017; Zhu et al., 2019). Except for cabbage (*B. oleracea* L. var. *capitata* L.) and turnip (*B. rapa* L. subsp. *rapa*), the total GSLs, glucoraphanin, glucoraphenin, glucobrassicin, glucotropeolin, gluconasturtiin, and glucobrassicanapin contents of rapeseed (*B. napus* L.), red radish (*Raphanus sativus* L.), and *Cardamine hirsuta* also increased after treatment with JA (Bae et al., 2014; Thiruvengadam and Chung, 2015; Thiruvengadam et al., 2016a; Bakhtiari et al., 2018). Transcriptome profiling and gene expression analysis indicated that the expression level of *MYB34*, *MYB51*, *MYB122*, and the gene expression of the indole core biosynthesis genes, including *CYP79B2*, *CYP83B1, CYP79F1, UGT74B1*, and *SOT16* increased in broccoli (*B. oleracea* L. var. *italica*) (Ku et al., 2016; Thiruvengadam et al., 2016b). JA also induced aliphatic GSL biosynthesis in rapeseed (*Brassica napus* L.), the levels of *AP2*/*ERF*, *bHLH*, *WRKY*, and *MYB*, which regulate the biosynthesis of total GSLs and aliphatic GSLs were also upregulated (Zhou and Memelink, 2016; Li et al., 2018). Proteins related to GSL biosynthesis and degradation were mediated by JA, leading to the accumulation of GSLs and sulforaphane in broccoli (*B. oleracea* L. var. *italica*) sprouts and the biosynthesis of indole GSLs in *A. thaliana* (Guo et al., 2017; Castillo et al., 2019).

Methyl jasmonate (MeJA), a kind of cyclopentanone derivative signal substance that exists widely in plants, can regulate plant growth, development, secondary metabolism, and disease resistance (Vahid and Soheil, 2019; Kurowska et al., 2020). MeJA application leads two and fourfold increases of 2-phenylethyl GSL and indole GSLs contents in turnip (*B. rapa* L. subsp. *rapa*) roots and leaves. Meanwhile, the 4-methoxy-3-indolylmethyl, 1-methoxy-3-indolylmethyl, 3-indolylmethyl, 2-phenylethyl, and indole GSLs contents in the root increased (Schreiner et al., 2011). Same with Schreiner et al. (2011) and Zang et al. (2015a) also concluded that roots accumulated much more GSLs and were more sensitive and rapidly responsive than leaves; indole GSLs (glucobrassicin, 4-methoxy glucobrassicin, and neoglucobrassicin) were the major component of total GSLs that accumulated rapidly in both roots and leaves (Zang et al., 2015a). Meanwhile, preharvest MeJA treatment increased 4- and 12-fold of GSL concentration in pak choi (*B. rapa* L.) in the soil and hydroponics-growing conditions, respectively (Baek et al., 2021). MeJA also significantly increased the concentrations of glucobrassicin, neoglucobrassicin, and indole GSLs in Chinese kale (*Brassica alboglabra*), and leaf-spraying MeJA induced greater accumulation of indole GSL (Sun et al., 2012b). Similar with turnip (*B. rapa* L. subsp. *rapa*) and kale (*B. oleracea* var. *acephala* DC), the glucoraphanin, glucoraphanin, and glucobrassicin contents in MeJA-treated China rose radish (*R. sativus* L. cv. China rose), red radish (*R. sativus* L. cv. Rambo), and *A. thaliana* was also enhanced (Bae et al., 2014; Chen et al., 2017). The contents of glucoraphenin, 4-hydroxyglucobrassicin, and glucobrassicin in radish (*R. sativus* L.) seedlings increased with increasing concentrations of MeJA (Al-Dhabi et al., 2015). After MeJA treatment, the glucobrassicin, 4-methoxyglucobrassicin, neoglucobrassicin, gluconasturtiin, and total GSLs concentrations in broccoli (*B. oleracea* L. var. *italica*) floret increased (Kang and Juvik, 2011; Ku and Juvik, 2012; Kang et al., 2013; Hassini et al., 2017a,b; Chiu et al., 2019, 2020). The accumulation of glucobrassicin, neoglucobrassicin, and gluconasturtiin in broccoli (*B. oleracea* L. var. *italica*) flower bulb (Liu et al., 2014; Ku et al., 2016), indole GSL content in broccoli (*B. oleracea* L. var. *italica*) suspension-cultured cells also enhanced (Sánchez-Pujante et al., 2020). Moreover, foliar application of MeJA + NaCl provoked higher GSL content than NaCl application alone (Rios et al., 2021). These results indicated that MeJA has different effects on aboveground leaves and roots, and the MeJA and NaCl may synergistically participate in the regulation of GSL biosynthesis.

The JA and MeJA are the most commonly used elicitors for induction of GSL. JA plays an important role in sensing and transmitting signals (Kim et al., 2021). JA regulation of GSL depends on the jasmonoyl-L-isoleucine-mediated COI1/JAZ/MYC2 pathways. When endogenous JA is below the threshold concentration, *JAZ* binds to *MYC* and prevents its transcription, thus inhibiting the expression of JA-responsive genes (Major et al., 2017). Under stress, JA content increases and isomerizes into JA-ILE, which promotes the interaction between *COI1* and *JAZ*, leading to degradation of *JAZ* protease and release of *MYC*, and promotes the expression of JA-responsive genes. After *MYC* release, regulation of *R2R3-MYB* transcription factor expression ultimately activates the GSL synthesis gene (Wasternack and Strnad, 2019; Kim et al., 2021). JA can be converted to the more volatile MeJA; MeJA also can be converted to JA by esterases; both JA and MeJA have been reported to function as transported signals that induce systemic wound responses (Stratmann, 2003; Sun and Zhang, 2021). MeJA can act as an elicitor to enhance GSL biosynthesis in leaves and roots by upregulating the *MYB*, *CYP79*, *CYP83*, *AOP2*, *FMOGS-OX5*, and other GSL biosynthesis-related genes (Kang et al., 2013; Chen et al., 2015; Yi et al., 2016; Yu-Chun et al., 2018). Thus, long-distance transport of GSLs between the roots and leaves through phloem and xylem vascular tissues could be possible depending on the type of GSLs (Zang et al., 2015b).

### Salicylic Acid

Salicylic acid (SA) is a small phenolic substance that exists widely in higher plants. It is a common endogenous signaling molecule in plants and plays important roles in physiological processes, such as disease, drought, cold, and salt resistance (Henschel et al., 2020; Xiao et al., 2020; Zhang et al., 2020). By examining the effects of SA and SA + JA in *A. thaliana*, Traw et al. (2003) and Barickman et al. (2013) found that SA could enhance the biosynthesis of GSLs but attenuated the induction of GSLs by JA, suggesting cross-effects between SA and JA (Traw et al., 2003; Barickman et al., 2013). SA also led to the enhancement of indole GSLs (glucobrassicin, 4-methoxy glucobrassicin, and neoglucobrassicin), aliphatic GSLs, and total GSLs in Chinese kale (*B. alboglabra*) roots and leaves. Compared with irrigation, leaf spraying produced higher amounts of indole GSLs (Sun et al., 2012b; Guo et al., 2013a; Zang et al., 2015a). Similar with Chinese kale, SA also led to the accumulation of the aromatic 2-phenylethyl, and different GSL contents in cabbage (*B. oleracea* L. var. *capitata* L.) and turnip (*B. rapa* L. subsp. *rapa*) (Schreiner et al., 2011; Thiruvengadam et al., 2016a; Yi et al., 2016).

Under abiotic stress, SA has a complex effect on the regulation of GSL and interacts with JA in the regulation of GSL biosynthesis (Guo et al., 2013a). ROS formation has a dual function, toxic to cells at high levels, and activate local and systemic defense responses to stress at low concentrations (You and Chan, 2015; Liu et al., 2021). ROS produces and amplified signals and then transmitted to SA, which regulates the expression of *R2R3-MYB* transcription factors through the MAPK cascade pathway (Liu et al., 2021). By enhancing the expression levels of *MYBs*, *CYP79F1* and *CYP83B1*, the GSL contents increased (Schreiner et al., 2011; Thiruvengadam et al., 2016a; Yi et al., 2016).

### Brassinosteroids and Ethylene

As a newly discovered plant endogenous hormone, brassinosteroids (BR) is internationally recognized as the most effective, broad-spectrum, non-toxic plant growth hormone, which cannot only regulate the photosynthesis, respiration, transpiration of plants but also improves stress resistance (Guo J. et al., 2018; Maghsoudi et al., 2019). Two studies on BR regulation of GSLs both focused on alleviating NaCl stress. Treated with different concentrations of BR, Guo et al. (2014) found that the GSL content in broccoli (*B. oleracea* L. var. *italica*) sprouts varied with the concentrations of BR. The contents of total GSLs and glucoraphanin increased by 86 and 85% after treatment with 2 nM BR under 40-mM NaCl stress (Guo et al., 2014). Similar to broccoli (*B. oleracea* L. var. *italica*), the 100-nM BR enhanced 1.23 ∼ 5.30-fold of glucoiberin, glucoraphanin, glucoerucin, gluconapin, progoitrin, sinigrin, total aliphatic GSLs, and total GSLs in Chinese kale (*B. alboglabra*) sprouts under 160-mM NaCl stress. Meanwhile, the expression levels of indole GSLs biosynthesis genes *MYB51* increased by fourfold. However, under normal cultivation conditions, the total GSLs content reduced 70.1% after 100-nM BR treatment, which indicates that BR may synergistically participate in GSL biosynthesis with NaCl (Wang et al., 2020).

Ethylene (ET) is a gaseous metabolite found in plants, which can inhibit plants growth and promotes leaf loss and fruit ripening (Bleecker and Kende, 2000). Sun et al. (2012a) concluded that there were no significant changes in the content of individual and total contents of aliphatic and indole GSLs in Chinese kale (*B. alboglabra*) after Ethrel vapor fumigation (Sun et al., 2012b). However, Thiruvengadam et al. (2015b) found that the ET significantly increased the contents of indole, aliphatic, and aromatic GSLs in rapeseed (*B. napus* L.) (Thiruvengadam et al., 2015b). As an inhibitor of ethylene, 1-methylcyclopropene (1-MCP) inhibited the decrease of total GSLs, sulforaphane, glucoraphanin, glucobrassicin in Chinese kale (*B. alboglabra*), broccoli (*B. oleracea* L. var. *italica*), and cabbage (*B. oleracea* L. var. *capitata* L.) (Yuan et al., 2010; Sun et al., 2012a; Kang et al., 2013; Xu et al., 2013).

The BRs function through a complex signal transduction pathway involving the BR receptor *BRI1* and its co-receptor BAK1. In addition, the BR signaling components *BZR1* and *BES1* are important transcription factors involved in the BR-signaling pathway that participates in the regulation of GSL biosynthesis by BR. BR signaling affects GSL metabolism *via* transcriptional and/or post-translational modifications of compounds in the GSL catabolic pathways (Lee et al., 2018; Wang et al., 2020). Plant responses are coordinated by several signaling systems. One of these, the oxylipin-signaling pathway includes the JA and related compounds, and has been shown to influence the production of various metabolic defenses, including GSL (Howe and Jander, 2008; Erb et al., 2012). ET pathways, which are one of the JA-signaling pathways, cross-communicate with other hormonal pathways, and the impact of ET signaling may involve in GSL biosynthesis mechanisms (Erb et al., 2012; Kang et al., 2013).

### Auxins and Gibberellic Acid

Auxins are the first discovered growth-promoting hormone in plants. Using different concentrations of indole−3−acetic acid (IAA) to treat hairy root culture of broccoli (*Brassica oleracea* L. var. *capitata* L.), Kim et al. (2013) concluded the accumulation of total GSLs, glucoraphanin, gluconapin, 4-hydroxyglucobrassicin, glucoerucin, glucobrassicin, 4-methoxyglucobrassicin, gluconasturtiin, and neoglucobrassicin, increased after 0.1-mg/L IAA treatment (Kim et al., 2013). Similar to IAA, 0.1-mg/L indolebutyric acid (IBA) and naphthylacetic acid (NAA) also resulted the highest accumulation of the total GSLs and the contents of eight individual GSLs (glucoraphanin, gluconapin, 4-hydroxyglucobrassicin, glucoerucin, glucobrassicin, 4-methoxyglucobrassicin, gluconasturtiin, and neoglucobrassicin) (Kim et al., 2013).

As a widely used hormone in plant production, gibberellic acid (GA_3_) can improve the growth, germination, flowering, and yield, as well as enhance plant-disease resistance (Maurya et al., 2019; Abbasi et al., 2020). Miao et al. (2017) found the GA_3_ treatment did not exert a remarkable influence on the aliphatic and total GSLs contents but increased the glucobrassicin Chinese kale (*B. alboglabra*) sprouts. However, combined treatment of GA_3_ and glucose increased the total GSLs content, indicating that GA_3_ and glucose synergistically regulate the biosynthesis of GSL (Miao et al., 2017).

It has recently been shown that the cytochromes *CYP79B2* and *CYP79B3* metabolize tryptophan to indole-3-acetaldoxime. This metabolite is often suggested to be the precursor of indole-3-acetonitrile (IAN) in IAA biosynthesis as well as the precursor of thiohydroximates in GSL biosynthesis. *CYP83B1* is a regulator of auxin production by controlling the flux of indole-3-acetaldoxime into IAA and indole GSL biosynthesis (Bak et al., 2001; Grubb et al., 2004; Malka and Cheng, 2017; Salehin et al., 2019). In addition, *CYP83B1* catalyzes the first committed step in indole GSL biosynthesis by metabolizing indole-3-acetaldoxime to its corresponding aci-nitro compound. Furthermore, the phylogenetic relationship between *CYP83B1* and *CYP71E1*, the cytochrome P450 involved in the oxime-metabolizing step in cyanogenic glucoside biosynthesis, argues for an evolutionary relationship between IAA, GSL, and cyanogenic glucoside biosynthesis (Bak et al., 2001; Grubb et al., 2004; Salehin et al., 2019).

### Abscisic Acid

Unlike other plant hormones, abscisic acid (ABA) could inhibit plant growth by promoting leaf shedding, causing bud dormancy and inhibiting cell elongation (Xie et al., 2020). Keling and Zhu (2013) concluded that 10-mg/L ABA decreased the proportion of aliphatic GSLs but increased the relative percentages of indole and aromatic GSLs in pak choi (*B. rapa* L.) shoots (Keling and Zhu, 2013). Meanwhile, the expression levels of the *MYB28*, *MYB51*, and *MYB122*, which are involved in indole and aliphatic GSL biosynthesis, were also negatively regulated in *A. thaliana* (Peskan-Berghofer et al., 2015). However, by application of different concentrations of ABA on cabbage (*B. oleracea* L. var. *capitata* L.) sprouts, the total GSLs content increased 72.7% under 50 μM (Wang et al., 2015). And indole GSLs (glucobrassicin, 4-methoxyglucobrassicin, neoglucobrassicin, and 4-hydroxyglucobrassicin) and aromatic GSL (gluconasturtiin) contents were significantly increased in turnip (*B. rapa* L. subsp. *rapa*) (Thiruvengadam et al., 2015b,2016a). The reasons for the discrepancies among the results of these studies might be the differences in the concentration of ABA treatments and the experimental species.

As a stress hormone, ABA content increases rapidly under drought, cold, high temperature, salt, and waterlogging. These abiotic stresses increase ABA delivery to guard cells, and the hydrolysis of GSLs catalyzed by myrosinases is also induced in some manner by ABA (Zhi et al., 2008). Meanwhile, ABA enhanced the expression levels of *MYB, CYP79F1*, and *CYP83B1*, and several sulfotransferase homologs in steps of the GSL core formation (Thiruvengadam et al., 2015b,2016a; Miret et al., 2017).

### Other Plant Growth-Regulating Substances

Studies have shown that, in addition to JA, MeJA, SA, EBR, and melatonin, certain other substances also can affect the synthesis of GSLs. Melatonin (MT) is an indole substance found in many organisms that, in plants, promotes seed germination, plant growth, and adventitious root formation (Chen et al., 2020; He and He, 2020). About 100-μM MT increased the total GSLs, sulforaphane, and glucoraphanin contents in postharvest broccoli (*B. oleracea* L. var. *italica*) (Wei et al., 2019); 1-mM MT also sustained higher content of GSLs and glucoraphanin in broccoli (*B. oleracea* L. var. *italica*) florets (Miao et al., 2020). Transcriptomics analysis showed the expression levels of glucoraphanin biosynthesis-related genes increased under MT treatment in broccoli (*B. oleracea* L. var. *italica*) hairy roots (Tian et al., 2021). Melatonin also enhanced the biosynthesis of cabbage (*B. oleracea* L. var. *capitata* L.) GSL and increased the GSL contents (Teng et al., 2021). These results showed that the MT could enhance the glucoraphanin content, and the different organs had different melatonin response concentrations.

6-benzylaminopurine (6-BAP) inhibited the decrease rate of total GSLs content in harvested broccoli (*B. oleracea* L. var. *italica*) florets (Xu et al., 2011), and 200 mg/L6-BA + 2.5 μL/L1-MCP enhanced the biosynthesis of GSLs and the formation of the sulforaphane (Xu et al., 2013). 5-aminolevulinic acid (ALA) increased the aliphatic, aromatic, and total GSLs contents, while the indole GSLs content (glucobrassicin, 4-methoxyglucobrassicin, and 1-methoxyglucobrassicin) decreased by regulating the expression level of the *UGT79B1*, *MYB12*, and *MYB28* in rapeseed (*B. napus* L.) seedlings (Maodzeka et al., 2019). Folic acid and coronatine effectively increase the level of GSLs in the post-harvested broccoli (*B. oleracea* L. var. *italica*) and suspension-cultured cells (Sánchez-Pujante et al., 2020). After liquiritin treatment, the total GSLs, 4-hydroxyglucobrassicin, 4-methoxyglucobrassicin, glucobrassicin, progoitrin, glucoraphanin, glucoiberin, neoglucobrassicin, and sinigrin contents significantly increased by enhancing the expression levels of genes involved in cabbage (*B. oleracea* L. var. *capitata* L.) GSL biosynthesis (Akram et al., 2020). Due to the lack of research reports on these substances, the mechanism of their regulation of GSL remains unclear.

## Metal Ions

### Selenium

As a rare element, selenium (Se) plays an important role in maintaining plant growth and development, promoting stress tolerance, enhancing resistance, and improving the quality index of cruciferous vegetables (Barickman et al., 2013; Azizi et al., 2020; Yin et al., 2020). SeO_2_ increased the amount of gluconasturtiin, glucobrassicanapin, glucoallysin, glucobrassicin, 4-methoxyglucobrassicin, and 4-hydroxyglucobrassicin rapeseed (*B. napus* L.) (Thiruvengadam and Chung, 2015). Na_2_SeO_3_ increased the formation of cabbage (*B. oleracea* L. var. *capitata* L.) indole-3-carbinol and indole-3-acetonitrile (Penas et al., 2012); the total GSLs and the sinigrin contents also increased exposure to Na_2_SeO_3_ alone or in combination with NaCl (Sun et al., 2018). Besides, the glucoraphanin, glucobrassicin, 4-methoxy-glucobrassicin, and total indole GSLs contents were significantly affected (Gao et al., 2021), while the aliphatic GSLs (glucoraphanin and glucoerucin) were not affected by Na_2_SeO_3_ in broccoli (*B. oleracea* L. var. *italica*) sprouts (He et al., 2020). Same with SeO_2_ and Na_2_SeO_3_, Na_2_SeO_4_ also significantly increased the expression of GSL biosynthesis genes in broccoli (*B. oleracea* L. var. *italica*) florets (Kim and Juvik, 2011; Rao et al., 2021). However, total GSLs contents in *Eruca sativa Mill* and *Diplotaxis tenuifolia* decreased after Na_2_SeO_4_ treatment (Dall’Acqua et al., 2019). Meanwhile, the glucoraphanin, glucocheirolin, glucoerucin, dimeric-4-mercaptobutyl, glucosativin, and neoglucobrassicin contents of *Eruca S. Mill* and *D. tenuifolia* (Dall’Acqua et al., 2019), and total GSL in radish (*R. sativus* L.) (Mckenzie et al., 2019) did not increase significantly. Total GSLs and sulforaphane levels were also not significantly influenced in broccoli (*B. oleracea* L. var. *italica*) sprouts between Na_2_SeO_3_ and Na_2_SeO_4_ treatments (Tian et al., 2016; Mahn, 2017). These studies indicated that different plant species, ages, and anions could lead to different biosynthesis modes of GSL.

Because of the similar structure of Se and sulfur (S), the absorption and transport modes of these two mineral elements in plants are basically the same, which leads to an antagonistic relationship between Se and S. Se is often serves as a substitute for S in physiological and metabolic processes in plants (Barickman et al., 2013). Se increases S uptake by preventing its downregulation at the plant’s roots; increasing Se helps S uptake into the plants more than increasing S fertilizer concentrations alone (Toler et al., 2007), and then affects the biosynthesis of GSLs by inducing the expression level of *MYBs*, *CYP79F1*, and *CYP83B1* (Sepuveda et al., 2013; Thiruvengadam and Chung, 2015; Mckenzie et al., 2019).

### Calcium and Zinc

Calcium is an essential nutrient element in plants and plays a central role in plant growth, development, and the response to environmental stress (Gao et al., 2020; Yoshioka and Moeder, 2020). Environmental stress enhances the Ca^2+^ content in plant tissues and mitigates adverse effect of plant (Sarker and Oba, 2018e; Sarker et al., 2018). Aliphatic GSLs (glucoerucin, glucoiberin, glucoiberverin, glucoraphanin, pentyl-GSL, and hexyl-GSL), indole GSLs (glucobrassicin, neoglucobrassicin, and 4-hydroxyglucobrassicin) (Sun et al., 2015), and sulforaphane contents in broccoli (*B. oleracea* L. var. *italica*) sprouts also increased significantly after the CaCl_2_ treatment (Yang et al., 2015b). And with an increase in the CaCl_2_ concentration, the total GSLs content increased first and then decreased (Yang et al., 2016). Similar to CaCl_2_, after preharvest CaSO_4_ treatment, the total GSLs, glucoraphanin, glucoerucin, glucobrassicin, and 4-hydroxyglucobrassicin contents increased significantly (Guo L. et al., 2018).

As one of the essential trace elements in plants, Zinc (Zn) is not only involved in the formation of auxin but also is a component and an activator of many enzymes (Atsushi et al., 2020). Zn has extensive effects on carbon and nitrogen metabolism, photosynthesis, and stress resistance of plants. In addition, Zn affects protein synthesis; therefore, in agricultural production, Zn supplementation is becoming increasingly popular. Low concentration of ZnSO_4_ and ZnCl_2_ increased the GSL contents in white cabbage (*B. oleracea* var. *capitata* f. alba) (Kusznierewicz et al., 2012), broccoli (*B. oleracea* L. var. *italica*) sprouts (Yang et al., 2015a; Rivera-Martin et al., 2021), and *Noccaea caerulescens* (Fones et al., 2019), turnip (*B. rapa* L. subsp. *rapa*) (Aghajanzadeh et al., 2020), and pak choi (*B. rapa* L.) (Fatemi et al., 2020).

Calcium plays an important role in regulation plant growth and signal transduction as well as the accumulation of secondary metabolites in plants under either natural or stress conditions (Medvedev, 2018; Iqbal et al., 2020). In the present study, Ca^2+^ is important in enhancement of GSL content during plant growth and storage (Yang et al., 2016). Ca^2+^ increased the formation of ITCs and GSL through indirect effects instead of acting on MYR activity during hydrolysis (Guo L. et al., 2018). In the tolerance range of Zn stress, Zn increased the accumulation of indole GSL and enhanced the antioxidant capacity and defense capacity of plants (Aghajanzadeh et al., 2020). Meanwhile, the biosynthetic precursor of GSL was increased by promoting the formation of cysteine (Cys) through the sulfur assimilation pathway (Aghajanzadeh et al., 2020), and the expression levels of indole GSL biosynthesis-related genes (*MYB34*, *CYP79B3*, and *CYP83B1*) were also significantly increased (Yang et al., 2015a).

### Cadmium and Arsenic

Cadmium (Cd), as one of the most harmful heavy metals, causing serious soil pollution, is not an essential element for plant growth. Cd can cause harm to plants at low concentrations, such as reducing the activity of enzymes and photosynthetic intensity, causing metabolic disorders, changing membrane permeability, preventing root growth, inhibiting root absorption of water and nutrients, and decreasing crop yield and quality (Hayashi et al., 2020; Jocsak et al., 2020). Therefore, it is of great scientific significance to study the effects of Cd on plant growth and development, and the tolerance mechanism of plants. CdCl_2_ and CdSO_4_ significantly decreased the total GSLs concentration in the *A. thaliana, Thlaspi praecox, Thlaspi arvense*, cabbage (*B. oleracea* L. var. *capitata* L.), and kale (*B. oleracea* var. *acephala* DC) (Roser et al., 2006; Sun et al., 2009; Kusznierewicz et al., 2012; Jakovljevic et al., 2013). Glucoibervirin and 4-methoxyglucobrassicin levels decreased significantly in the CdCl_2_-treated leaves, while the glucobrassicin, neoglucobrassicin, and 4-methoxyglucobrassicin levels all showed significant decreases in the *A. thaliana* roots (Sun et al., 2009). Similarly, a decrease in both aliphatic and indole GSLs contents associated with an increase in CdCl_2_ accumulation was observed in the roots and shoots of rapeseed (*B. napus* L.) plantlets under *in vitro* sterile conditions, demonstrating that Cd stress has a highly significant effect on roots’ and shoots’ GSL biosynthesis (Durenne et al., 2018). Different from *A. thaliana* and rapeseed (*B. napus* L.), CdCl_2_ had considerably enhanced gluconasturtiin and 4-hydroxyglucobrassicin levels in turnip (*B. rapa* L. subsp. *rapa*). Moreover, genes related to GSLs showed significant induction (Thiruvengadam and Chung, 2015).

As plants grow, they absorb arsenic from the environment, either passively or actively, causing damage to plants (Samanta et al., 2021). By examining two mustard (*Brassica juncea*) cultivars under different concentrations of Arsenic (As) stress, Pandey et al. (2016) concluded the As stress reduced the amount of total GSLs, and the GSLs content decreased with the increase of As concentration. Meanwhile, the overall contents of total aliphatic and indole GSLs decreased (Pandey et al., 2016).

Cadmium stress inhibits organic sulfur from entering the GSL synthesis pathway, promotes the biosynthesis of GSH and phytochelatins (PC) to detoxification, and enhances the tolerance mechanism of plants (Jakovljevic et al., 2013; Durenne et al., 2018). Under cadmium stress, plants can reduce the content of indolyl methyl 3-GSL, promote the generation of IAA, and regulate root growth (Jakovljevic et al., 2013; Durenne et al., 2018). After Cd^2+^ stress, cells can activate mitogen-activated protein kinase (MAPK), Ca^2+^/calmodulin system (Ca^2+^/CaM), JA, SA, and other stress response signal molecules. Then, these signal molecules fuse to regulate the family of transcription factors such as MYB in the nucleus (Xu et al., 2015).

### Cuprum and Ag NPs

Cuprum (Cu) is not only a component of various enzymes but is also closely related to carbon assimilation, nitrogen metabolism, absorption, and redox processes in plants (Letchumanan et al., 2021; Yusuf et al., 2021). At the same time, it is beneficial to the growth and development of crops and can affect the photosynthetic capacity and drought- and cold-resistance ability. Drought stress augmented the Cu content in plant tissues and mitigated the adverse effect of plants (Sarker and Oba, 2018d). High concentrations (50 ∼ 500 mM) CuSO_4_ decreased the turnip (*B. rapa* L. subsp. *rapa*) GSL content (Jahangir et al., 2008). The GSL content of *Nasturtium officinale* also decreased under 0.1 ∼ 1-mM CuCl_2_ (Kim J. et al., 2018). Under CuCl_2_ stress, the content of total GSLs, and indole and aromatic GSLs were only elevated in the Chinese cabbage [*Brassica pekinensis* (Lour.) Rupr.] roots. By enhancing the transcript levels of *CYP79B2*, *CYP83B1*, and *MYB51* in GSL biosynthesis, the indole GSLs and glucobrassicin levels increased by two and fourfold under treatment with 5- and 10-μM CuCl_2_, respectively. In addition, the indole GSLs in the roots could be considered as an index to judge effects of the CuCl_2_ concentrations (Aghajanzadeh et al., 2019).

Currently, research on the effect of Argentum (Ag) on the synthesis of GSLs has mainly focused on silver nanoparticles (AgNPs). In turnip (*B. rapa* L. subsp. *rapa*), glucoallysin, glucobrassicanapin, sinigrin, progoitrin, gluconapin, glucobrassicin, 4-methoxyglucobrassicin, 4-hydroxyglucobrassicin, neoglucobrassicin, and gluconasturtiin contents, and the levels of their associated transcription factors (*MYB28*, *MYB29*, *MYB34*, and *MYB51*) enhanced significantly after biologically synthesized AgNP treatment (Thiruvengadam et al., 2015a; Chung et al., 2018). The AgNPs also enhanced the total GSL content in *A. thaliana* (Kruszka et al., 2019; Zhang et al., 2019). Similar with AgNP, low concentration of AgNO_3_ (10 μM) enhanced the accumulation of glucosiberin and glucohirsutinin *N. officinale* (Kim J. et al., 2018).

Similar to Zinc, Cu can also enhance the accumulation of indole GSL within the tolerance range, and then enhance the antioxidant capacity and defense capacity of plants (Aghajanzadeh et al., 2019). The distribution of organic sulfur to GSL in plants under Cu stress was higher than that of organic sulfur compounds such as GSH, thus promoting the accumulation of GSL (Aghajanzadeh et al., 2019). Moreover, Cu stress promoted indole GSL biosynthesis by enhancing the expression of *MYB51*, *CYP79B2*, and *CYP83B1* (Kolbert et al., 2012; Aghajanzadeh et al., 2019). Ag can induce ROS production and subsequently increase the expression levels of MYB transcription factor, *SUR1*, and *ST5C*, thus inducing the signal transduction pathway of GSL accumulation (Thiruvengadam et al., 2015a; Chung et al., 2018).

## Salinity Stress

Salinity stress causes many physiological and molecular changes, such as imbalance in Na^+^ and K^+^ (Sarker and Oba, 2020), creation of reactive oxygen species (ROS) and oxidative stress (Sarker and Oba, 2018b), osmotic stress (Sarker and Oba, 2018c), which eventually interrupt the growth and productivity of crops (Sarker and Oba, 2018a,^2019^). Saline stress is mainly NaCl stress. In general, the GSL content increased under low and moderate salt stress; 40 ∼ 160-mM NaCl increased total GSL, glucoerucin, glucobrassicin, sulforaphane, neoglucobrassicin, and 4-hydroxy glucobrassicin contents in broccoli (*B. oleracea* L. var. *italica*) (Carmen et al., 2008; Gioia et al., 2018; Rios et al., 2020). Glucoalyssin, gluconapin, glucobrassicin, and neglucobrassicin contents in pak choi (*B. rapa* L.) enhanced under 50-mM NaCl (Hu and Zhu, 2010). In cabbage (*B. oleracea* L. var. *capitata* L.), the total GSLs increased (Sun et al., 2018); the aliphatic and indole GSLs increased 1.29- and 1.42-fold, respectively (Wang et al., 2020). And the GSL contents of Chinese cabbage [*Brassica pekinensis* (Lour.) Rupr.], cabbage (*B. oleracea* L. var. *capitata* L.), kale (*B. oleracea* var. *acephala* DC), and *E. sativa* were elicited by NaCl in a dose-dependent manner (Cocetta et al., 2018; Linic et al., 2019; Samec et al., 2021), and different rapeseed (*B. napus* L.) genotypes also showed varying levels of GSL accumulation (Gyawali et al., 2019). However, the GSL content decreased under high NaCl concentrations in red radish (*R. sativus* L.) (<500 mM) (Chen et al., 2018), and *D. tenuifolia* (130 mM) (Petretto et al., 2019). KCl, Na_2_SO_4_, K_2_SO_4_, and other salts can also affect the biosynthesis of GSL. About 50 ∼ 100-mM KCl did not affect the total GSLs, the GSL composition in the roots, whereas it resulted in an up to 60% decrease in total GSLs content of the shoot in turnip (*B. rapa* L. subsp. *rapa*) (Aghajanzadeh et al., 2018). The total and individual GSLs in the root increased 1.8 ∼ 4.5-fold in the 50-mM Na_2_SO_4_, while 50-mM K_2_SO_4_ significantly increased the contents of indole and aromatic GSL in the shoots. Phosphate-sufficient plants exhibited lower GSL concentrations, phosphite increased the pak choi (*B. rapa* L.) 1-methoxyindol-3-ylmethyl content compared with low concentration of phosphite, while high phosphite levels increased the but-3-enyl-GSL concentration compared with the medium levels of phosphite in mustard (*B. juncea*) (Trejo-Tellez et al., 2019).

When salinity stress is within the tolerance level of plants, the increase of GSL content will participate in osmotic regulation to maintain water balance (Carmen et al., 2008), enhance plant-defense response, and maintain plant growth (Aghajanzadeh et al., 2018; Fatemi et al., 2020). However, high concentration of salinity stress can increase the activity of GSL degradation enzyme DtTMT, resulting in the decrease of GSL content. Plants will distribute more sulfur to the primary assimilation process, promote the glutathione (GSH) biosynthesis, thus limiting the GSL biosynthesis (Reich et al., 2017; Aghajanzadeh et al., 2018). At the same time, the enhanced ROS signal in chloroplast of plants under salt stress will further promote the transcription level of transcription factor gene *MYBs* and GSL biosynthetic genes (Sewelam et al., 2014; Pilarska et al., 2016; Aghajanzadeh et al., 2018; Wang et al., 2020).

## Glucose and Sucrose

In the process of plant growth, glucose (Glu) is generated by photosynthesis. When subjected to the stress of an adverse environment, the Glu generated cannot meet the growth and reproduction needs of the plants. Exogenous supplementation of Glu could increase plant-stress resistance (e.g., cold resistance and drought resistance), and promote growth and propagation. By exogenous Glu treatment, the glucoraphasatin, 4-OH-glucobrassicin, and 4-methoxyglucobrassicin contents dramatically reduced in Chinese kale (*B. alboglabra*) and radish (*R. sativus* L.) sprouts, while the content of gluconapin and glucobrassicanapin increased markedly (Wei et al., 2011). Total GSLs, glucoraphanin, glucoraphenin, and glucobrassicin contents of cabbage (*B. oleracea* L. var. *capitata* L.), rapeseed (*B. napus* L.), turnip (*B. rapa* L. subsp. *rapa*), and mustard (*R. sativus*) increased after treatment with 277-mM Glu (Bae et al., 2014). However, Glu did not influence the individual and total GSLs of the Chinese kale sprouts significantly (Miao et al., 2017). Glu + JA enhanced the *A. thaliana* GSL content significantly, whereas the synergistic effect of SA + Glu was less obvious (Guo et al., 2013a; Miao et al., 2013, 2016). The induction of indole and aliphatic GSLs was inhibited after treatment with JA and Glu. In addition, in the Glu-insensitive mutants, the effects of JA and Glu on the GSL content reduced significantly, suggesting that JA and Glu signaling is involved in cross-talk in regulating GSL biosynthesis. Moreover, Glu upregulates GSLs *via* the ABA signaling pathway and decreased accumulation of 4-methylthiobutyl (Li et al., 2021). Same with Glu, the contents of total GSLs, glucoiberin, glucoraphanin, glucobrassicin, sulforaphane, glucobrassicin, and neoglucobrassicin in cabbage (*B. oleracea* L. var. *capitata* L.), rapeseed (*B. napus* L.), turnip (*B. rapa* L. subsp. *rapa*), and radish (*R. sativus*) sprouts, and broccoli (*B. oleracea* L. var. *italica*) florets increased significantly and maintained a higher level after sucrose treatment (Guo et al., 2011; Bae et al., 2014; Xu et al., 2016), while the level of GSLs in fructooligosaccharides (FOS)-treated turnip (*B. rapa* L. subsp. *rapa*) decreased markedly (Thiruvengadam et al., 2016b).

Sugars play important roles in plant growth and development as a carbon and energy source. They can also act as effective signaling molecules throughout plant life (Bolouri-Moghaddam et al., 2010; Smeekens et al., 2010). Hexokinases (HXKs) are one of the most conserved sugar sensors together with other sugar kinases, and carry out diverse and distinct functions in glucose metabolism and signaling (Rolland et al., 2002; Miao et al., 2013). Among them, HXK1-dependent glucose signaling can affect plant growth, which relies on the endogenous glucose level and the sensitivity to glucose. By regulating the HXK1-mediated signaling, the *MYB* transcription factors expression levels, which participated in the regulation of GSL biosynthesis, changed (Fritz et al., 2010; Guo et al., 2013a; Miao et al., 2013).

## Environmental Pollution

### Gaseous Contamination

Gaseous contamination (SO_2_, H_2_S, and O_3_) is a very serious environmental problem affecting crop growth, which can lead to changes in the biosynthesis of bioactive molecules (Mukherjee et al., 2019). By determining the shoots and roots, GSL contents treated with different sulfur sources (i.e., sulfate, sulfite, and sulfide), SO_2_, and H_2_S. Aghajanzadeh et al. (2014, 2015) concluded that sulfate deprivation resulted in a strong decrease in the content and an altered composition of the mustard (*B. juncea*) and turnip (*B. rapa* L. subsp. *rapa*) GSLs. H_2_S and SO_2_ did not affect the total content but slightly affected the GSL composition in the shoots and roots of turnip (*B. rapa* L. subsp. *rapa*). Meanwhile, SO_2_ and H_2_S exposure largely alleviated the decrease in C4-aliphatic GSLs (glucoerucin, gluconapin, and progoitrin) in roots of turnip (*B. rapa* L. subsp. *rapa*) and mustard (*B. juncea*) (Aghajanzadeh et al., 2014, 2015). Different from SO_2_ and H_2_S, O_3_ pollution can inhibit the accumulation of GSL. The total GSLs contents in rapeseed (*B. napus* L.) (Gielen et al., 2006; Himanen et al., 2008), black mustard (*B. nigra*) (Khaling et al., 2015), turnip (*B. rapa* L. subsp. *rapa*) (Han et al., 2020, 2021) decreased, while the accumulation of aromatic GSL increased (Han et al., 2020, 2021).

Greenhouse effect leads to the increase of CO_2_ content in the air, which then affects GSL on plants. High CO_2_ concentration significantly increased the contents of aromatic GSL in cabbage (*B. oleracea* L. var. *capitata* L.) (Reddy et al., 2004), and rapeseed (*B. napus* L.) leaves (Himanen et al., 2008), but had no significant effect on the total amount of GSL. However, as CO_2_ treatment enhanced primary production, the total GSLs contents of broccoli (*B. oleracea* L. var. *italica*) (Schonhof et al., 2007; Rodriguez-Hernandez et al., 2014; Almuhayawi et al., 2020), Chinese kale (*B. alboglabra*) (La et al., 2009), Brussels sprout (*B. oleracea* var. *gemmifera*) (Klaiber et al., 2013), *A. thaliana* (Paudel et al., 2016), kale (*B. oleracea* var. *acephala* DC) (Chowdhury et al., 2021), and the accumulation of glucoraphanin and sulforaphane also increased in broccoli (*B. oleracea* L. var. *italica*) sprouts (Almuhayawi et al., 2020). Thus, it is conceivable to recycle excess CO_2_ by using it as supplement greenhouse gas to produce high-GSL cruciferous plants (Wiesner-Reinhold et al., 2021).

Accumulation of aromatic and indole GSL under O_3_ stress can enhance the mechanism of antioxidant damage and physical damage resistance in plants, and then induce the accumulation of JA and SA, which promote the biosynthesis of indole GSL and aromatic GSL (Han et al., 2021). Therefore, O_3_ regulates the biosynthesis of GSL by inducing the production of ROS and activating the JA and SA signal transduction pathways (Zhang et al., 2017). However, the influence of O_3_ stress on the transcription levels of GSL transcription factors and biosynthetic genes remains to be further studied. The pathway of GSL response under H_2_S stress has not been reported. CO_2_ induces the formation of amino acids and the precursors of GSL biosynthesis through different pathways. High concentrations of CO_2_ not only promote the biosynthesis of Cys (the precursor of Met) (Rodriguez-Hernandez et al., 2014) but also promote the accumulation of amino acids and other compounds (Rodriguez-Hernandez et al., 2014; Almuhayawi et al., 2020).

### Chemical Pollution

Pesticides are indispensable chemical agents in modern agricultural production to control diseases, insect pests, and weeds, and also play an important role in the yield and quality of crops (Huang et al., 2021; Zhang and Yang, 2021). However, the excessive use of pesticides causes great harm to the natural environment and induces pesticide stress, which affects the normal physiological and metabolic activities of crops. Imidacloprid, pyramezone, beta-cypermethrin, and acephate treatments significantly increased the contents of Pak Choi (*B. rapa* L.) total GSLs content and all individual GSL contents, especially the aliphatic group (Zhu et al., 2015). In fluridon (Flu)-treated sprouts, the cabbage (*B. oleracea* L. var. *capitata* L.) GSL content and isothiocyanate formation reduced by 46.5 and 38%, respectively (Wang et al., 2015). By increasing the content of amino acids of the precursor of GSL biosynthesis, pesticides can increase the content of GSLs in Pak Choi (*B. rapa* L.) (Zhu et al., 2015).

There are many inorganic and organic toxic substances in the environment that potentially affect plants (Thompson and Darwish, 2019). The contents of total GSLs and sulforaphane increased significantly after treatment with mannitol in broccoli (*B. oleracea* L. var. *italica*) (Guo et al., 2011). Putrescine treatment enhanced 4-hydroxyglucobrassicin and gluconasturtiin contents considerably in turnip (*B. rapa* L. subsp. *rapa*) (Thiruvengadam and Chung, 2015). Adenosine monophosphate (AMP) and gamma-aminobutyric acid (GABA) increased the amount of GSLs significantly in turnip (*B. rapa* L. subsp. *rapa*) (Thiruvengadam et al., 2016b). Using an RNA sequencing analysis, Gudio et al. (2018) detected the *CYP79B3* was misregulated and decreased the amount of *A. thaliana* indole GSLs in the presence of carbenicillin (Gudio et al., 2018).

## Future Work

**FIGURE 1:**
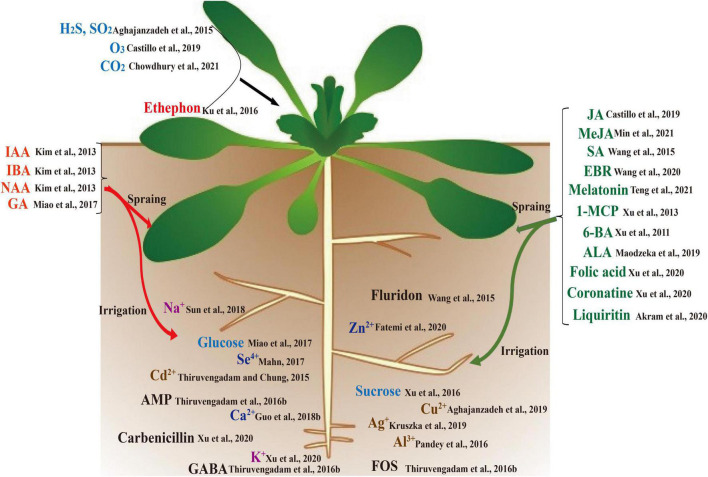
Chemicals that affect the biosynthesis of cruciferous glucosinolates (GSLs).

**FIGURE 2:**
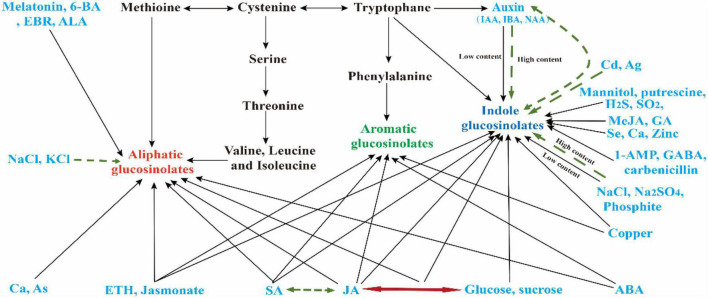
Relationship between different chemicals in GSLs biosynthesis. Figures in a blue font represent different exogenous chemicals; black and green single arrows indicate chemicals that can promote and inhibit the synthesis of GSL, respectively. Green-dotted-line double arrows represent the two substances showing antagonism in the synthesis of GSL, while the red double arrow indicates that the two substances coordinate to regulate GSL synthesis in Cruciferous crops.

As more biological functions of GSLs and their metabolites are gradually revealed in plant defense, human anti-cancer antibacterial, biological control, the regulation of GSL biosynthesis has become one of the hot topics in research into specialized metabolism and the stress response of cruciferous plants (Vo et al., 2018; Mitreiter and Gigolashvili, 2021; Sun and Zhang, 2021). Various researchers have carried out a large number of studies on the regulation mechanism of GSL synthesis. These studies have shown that there are many factors affecting the GSL biosynthesis and component content, including genotype, development stage, organs, and environmental conditions (Rhee et al., 2020; Chowdhury et al., 2021). Plant hormones, plant growth regulators, mineral elements, heavy metal, antibiotics, and other chemicals can also affect GSL biosynthesis (Figure 1) (Yuan et al., 2010; Kim et al., 2013; Zhou and Johan, 2017; Jocsak et al., 2020). Meanwhile, the content and composition of GSLs are closely related to the plant materials, plant organ/tissue, and the concentration of these chemicals (Bruinsma et al., 2007). Same concentration of a biological agent leads to different GSL compositions and response mechanisms in different plants, and the response mechanism of same chemicals in different periods is also different (Wang et al., 2015; Miret et al., 2017). Moreover, there are cross-talk effects among these chemicals (Figure 2). These studies provide data support and theoretical guidance for the use of exogenous chemicals to regulate the content of GSLs in the Cruciferae in subsequent production (Table 1). However, most of these studies focused on the changes in the GSL content induced by exogenous chemicals, and regulation at the transcriptional level, the specific upstream action elements, and regulation modes of transcription factors in the process of responding to exogenous chemicals require further research (Ku et al., 2014; Zhou and Memelink, 2016). Moreover, many studies have confirmed that GSL synthesis is regulated cooperatively by different hormones, chemicals, and environments; however, the interaction mechanisms among these substances are still unclear (Traw et al., 2003; Cipollini et al., 2010; Zang et al., 2015a) (Figure 2); that is, how the crop perceives the stimulation of exogenous hormones, growth regulators, or chemical substances, which pathways subsequently act on GSL synthesis, and how feedback regulates GSL synthesis after the stimulation disappears. Elucidation of this interaction mechanism will not only enrich the regulation network of GSL biosynthesis but also provide new ideas and methods to regulate the GSL biosynthesis by using chemical substances.

**TABLE 1:** Effects of different exogenous chemical substances on glucosinolates (GSL) synthesis in Cruciferous crops.

Substance	Concentrations	Component	Species	Correlation	References
**Chemical substances**					
Jasmonic acid (JA)	None; None	Total GSL; indole GSL	*A. thaliana*	Positive	Traw et al., 2003; Castillo et al., 2019
	0.1 mM; 200 μM; 1 mM	Sulforaphane; sinigrin, gluconapin, progoitrin, glucoiberin, total GSLs; 1-methoxyindol-3ylmethyl	(*B. oleracea* L. var. *capitata* L.)	Positive	Bruinsma et al., 2007; Wiesner et al., 2013a; Guo et al., 2017
	0.01 mM; 150μM	Glucoiberin, 4-hydroxyglucobrassicin; total GSLs, glucoraphanin, glucoraphenin, glucobrassicin	(*B. oleracea* L. var. *capitata* L.)	Negative	Bruinsma et al., 2007; Bae et al., 2014
	50 ∼ 200 μM	1-methoxyindol-3-ylmethyl	*Sinapis alba*	Positive	Kastell et al., 2013
	50 ∼ 200 μM; 150 μM	1-methoxyindol-3-ylmethyl; total GSLs, glucoraphanin, glucoraphenin, glucobrassicin; 4-methoxyglucobrassicin, neoglucobrassicin, 4-hydroxyglucobrassicin; aliphatic GSL, glucobrassicin	*B. rapa*	Positive	Kastell et al., 2013; Bae et al., 2014
	100 μM; 2.4 μM	Total GSLs; glucobrassicin, glucotropaeolin, gluconasturtiin, glucobrassicanapin	*B. rapa*; *C. hirsuta*	Positive	Thiruvengadam et al., 2015b; Bakhtiari et al., 2018
	150 μM; None	Aliphatic GSL	*B. napus*; *M. incana*	Positive	Bae et al., 2014; Li et al., 2018
	130 μM; 200 μM; 0.1 ∼ 0.2 μM; 0.5 μM	Indole GSL, 1-methoxy-indol-3-ylmethyl, aromatic 2-phenylethyl	*B. rapa*	Positive	Schreiner et al., 2011; Wiesner et al., 2013a; Zang et al., 2015a; Baek et al., 2021
	20 μM	Indole GSL, 1-methoxy-indol-3-ylmethyl, aromatic 2-phenylethyl	*A. thaliana*	Positive	Chen et al., 2017
	100 μM; 250 μM;	Total GSLs, glucoraphanin, glucoraphenin, glucoiberin, progoitrin, sinigrin, gluconasturtiin, glucobrassicin, neoglucobrassicin	*B. alboglabra*	Positive	Sun et al., 2012b; Yi et al., 2016
	250 μM; 250 μM; 250 mM; 100 μM; 250 μM	Total GSLs, glucoraphanin, glucoraphanin, glucoiberin, progoitrin, sinigrin, gluconasturtiin, glucobrassicin, neoglucobrassicin, glucoraphenin, glucoerucin, glucotropaeolin	*B. oleracea*	Positive	Kang and Juvik, 2011; Ku and Juvik, 2012; Kang et al., 2013; Al-Dhabi et al., 2015; Yi et al., 2016
Salicylic acid (SA)	100 μM; 2 mM	Aromatic 2-phenylethyl, indole, aliphatic and aromatic GSL; indole and total GSLs	*B. rapa*	Positive	Thiruvengadam et al., 2015b; Zang et al., 2015a
	1 ∼ 5 mM	4-methoxyglucobrassicin and aliphatic GSL	*B. alboglabra*	Positive	Sun et al., 2012b
	800 μM	Glucoallysin, sinigrin, progoitrin, gluconapin and glucobrassicanapin	*B. oleracea*	Positive	Yi et al., 2016
SA + JA	0.45 μM; none	Total GSLs	*A. thaliana*	Positive	Traw et al., 2003; Cipollini et al., 2010
EBR	20 nM; 100 nM	Total GSL, glucoraphanin; total GSL	*B. oleracea*; *B. alboglabra*	Positive	Guo et al., 2014; Wang et al., 2020
Ethephon	100 μM	Indole, aliphatic and aromatic GSL	*B. rapa*	Positive	Thiruvengadam et al., 2015b
	50 μM	Total aliphatic, indole GSLs	*B. alboglabra*	Positive	Sun et al., 2012b
IAA	0.1 mg/L	Glucoraphanin, gluconapin, 4-hydroxyglucobrassicin, glucoerucin, glucobrassicin, 4-methoxyglucobrassicin, gluconasturtiin, neoglucobrassicin	(*B. oleracea* L. var. *italica*)	Positive	Kim et al., 2013
IBA	0.1 mg/L	Total GSLs, glucoraphanin, gluconapin, gluconasturtiin, 4-hydroxyglucobrassicin, glucoerucin, glucobrassicin, 4-methoxyglucobrassicin, neoglucobrassicin	(*B. oleracea* L. var. *italica*)	Negative	Kim et al., 2013
NAA	0.1 mg/L	Total GSLs, glucoraphanin, gluconapin, glucoerucin, glucobrassicin, neoglucobrassicin, gluconasturtiin, 4-hydroxyglucobrassicin, 4-methoxyglucobrassicin,	(*B. oleracea* L. var. *italica*)	Positive	Kim et al., 2013
GA_3_	5 μM	Total GSL, aliphatic GSL, glucobrassicin	*B. alboglabra*	Positive	Miao et al., 2017
GA_3_ + glucose	5 μM + 30 g/L	Total GSLs, Glucoiberin, Progoitrin, Sinigrin, Glucoraphanin, Gluconapin, Glucoerucin, Glucobrassicin	*B. alboglabra*	Positive	Miao et al., 2017
ABA	10 μM; none	Total GSLs; 4-methoxyindol-3-ylmethyl-GSL	*A. thaliana*	Negative	Peskan-Berghofer et al., 2015; Hillwig et al., 2016
	50 mM; 10 μM	Total GSLs; total GSLs, isothiocyanate formation, myrosinase activity	*B. oleracea* L. var. *capitata* L.	Negative	Wang et al., 2015; Miret et al., 2017
	10 mg/L	Indole and aromatic GSL	*B. napus*	Positive	Keling and Zhu, 2013
	100 μM; 50 μM	Indole, aliphatic, aromatic GSL; gluconasturtiin	*B. rapa*	Positive	Thiruvengadam et al., 2015b; Wang et al., 2015
Melatonin	1 μM; 100 μM	Total GSLs; glucoraphanin, sulforaphane.	*B. oleracea* L. var. *italica*	Positive	Miao et al., 2020; Teng et al., 2021
6-BA	200 mg/L	Total GSLs; sulforaphane	*B. oleracea* L. var. *italica*	Positive	Xu et al., 2011
6-BA + 1-MCP	2.5 μM + 200 mg/L	Total GSLs, sulforaphane	*B. oleracea* L. var. *italica*	Positive	Xu et al., 2013
5-aminolevulinic acid (ALA)	0.5 ∼ 1 mg/L	Total GSLs	*B. napus*	Positive	Maodzeka et al., 2019
Folic acid	5 mg/L	Total GSLs	*B. oleracea* L. var. *italica*	Positive	Xu et al., 2020
Coronatine	0.5 μM	Total GSLs	*B. oleracea* L. var. *italica*	Positive	Sánchez-Pujante et al., 2020
Liquiritin	750 ppm	Total GSLs, progoitrin, sinigrin, glucoraphanin, glucobrassicin, glucoiberin, 4-methoxyglucobrassicin, neoglucobrassicin, 4-hydroxyglucobrassicin	*B. oleracea* L. var. *capitata* L.	Positive	Akram et al., 2020
**Metal ions**					
Selenium	5.2 mM; 0.3 μg/L; 25 μM; 20 μM;	Indole-3-carbinol; indole-3-acetonitrile; sinigrin; sulforaphane	*B. oleracea* L. var. *capitata* L.	Positive	Kim and Juvik, 2011; Penas et al., 2012; Thiruvengadam and Chung, 2015; Mahn, 2017
	100 μM; 2 mg/L	Glucoraphanin, gluconasturtiin; total GSLs	*B. oleracea* L. var. *capitata* L.	None	Tian et al., 2016; Sun et al., 2018
	25 μM	Gluconasturtiin, glucobrassicanapin, glucoallysin, glucobrassicin, 4-methoxyglucobrassicin, 4-hydroxyglucobrassicin	*B. rapa*	Positive	Thiruvengadam and Chung, 2015
	5 ∼ 20 μM; 10 ∼ 40 μM	Total GSLs, glucoraphanin, glucocheirolin, glucoerucin, dimeric-4-mercaptobutyl, glucosativin, neoglucobrassicin	*E. sativa*; *D. tenuifolia*	Negative/none	Dall’Acqua et al., 2019
Calcium	10 μM; 11 mM; 5 ∼ 15 mM; 10 mM	Sulforaphane, aliphatic (glucoerucin, glucoiberin, glucoiberverin, glucoraphanin, pentyl-GSL, and hexyl-GSL), indolic (glucobrassicin, neoglucobrassicin, 4-hydroxyglucobrassicin, glucoraphanin	*B. oleracea* L. var. *capitata* L.	Positive	Sun et al., 2015; Yang et al., 2015b,^2016^; Guo L. et al., 2018
Zinc	50 ∼ 200 μg/L; 2 mM; 10 μM; 25 mM	Total GSLs, glucoraphanin; total GSLs	*B. oleracea* L. var. *capitata* L.; *B. oleracea* L. var. *italica*; *B. rapa*	Positive	Kusznierewicz et al., 2012; Yang et al., 2015a; Aghajanzadeh et al., 2020; Fatemi et al., 2020
Cadmium	50 μM	Total GSLs, indole GSL, glucoibervirin, 4-methoxyglucobrassicin, neoglucobrassicin	*A. thaliana*	Negative	Sun et al., 2009
	25 μM; 5 ∼ 45 μM	Indole and aliphatic GSL/gluconasturtiin, 4-hydroxyglucobrassicin	*B. napus*	Negative/positive	Thiruvengadam and Chung, 2015; Durenne et al., 2018
Arsenic	150 ∼ 300 μM	Total GSLs, individual GSL content, aliphatic GSL	*B. juncea*	Positive	Pandey et al., 2016
Copper	5 ∼ 10 μM	Total GSLs, indolic and aromatic GSL	*B. napus*	Positive	Aghajanzadeh et al., 2019
Argentum	1 μM; 0.5 ∼ 5 ppm	Glucoallysin, glucobrassicanapin, sinigrin, progoitrin, gluconapin, 4-methoxyglucobrassicin, 4-hydroxyglucobrassicin, glucobrassicin, neoglucobrassicin, gluconasturtiin; total GSLs	*B. rapa*; *A. thaliana*	Positive	Chung et al., 2018; Kruszka et al., 2019
**Saline stress**					
NaCl	50 mM	Glucoalyssin, gluconapin, glucobrassicin, neoglucobrassicin gluconapin, glucobrassicin	*B. campestris*	Positive	Hu and Zhu, 2010
	100 mM; 160 mM	Glucoerucin, glucobrassicin, sulforaphane, 4-hydroxy glucobrassicin, neoglucobrassicin; total GSLs	*B. oleracea L.* var. *capitata* L.	Positive	Guo et al., 2013b; Gioia et al., 2018
	50 mM/L; 80 mM/L	Aliphatic GSL	*B. napus*	Negative	Aghajanzadeh et al., 2018; Sun et al., 2018
Phosphate	0.5 mM	Alkyl-GLs/alkenyl-GLs	*B. campestris*; *B. juncea*	Negative	Trejo-Tellez et al., 2019
Phosphite	0.5 mM	But-3-enyl-GSL, indol-3-ylmethyl-GSL	*B. campestris*; *B. juncea*	Positive	Trejo-Tellez et al., 2019
Glucose (Glu)	277 mM	Total GSLs, glucoraphanin, glucoraphenin, glucobrassicin	*B. oleracea*, *B. napus*, *B. rapa*, *R. sativus*	Positive	Bae et al., 2014
	50 mg/L	Glucoraphasatin, 4-OH-glucobrassicin, 4-methoxyglucobrassicin; gluconapin, glucobrassicanapin	*B. alboglabra*, *B. napus*	Positive	Wei et al., 2011
	None	Individual and total GSLs	*B. alboglabra*	None	Miao et al., 2017
Glucose + JA/Glu + SA	None; None	Total GSLs	*A. thaliana*	Positive/none	Guo et al., 2013a; Miao et al., 2013
Sucrose	176 mM; 146 mM; 12 g/L	Total GSLs, glucoiberin, glucoraphanin, glucobrassicin, sulforaphane, glucobrassicin, neoglucobrassicin	*B. oleracea*, *B. napus*, *B. rapa*, *R. sativus*	Positive	Guo et al., 2011; Bae et al., 2014; Xu et al., 2016
**Gaseous contamination**					
H_2_S, SO_2_	0.25 μL/L	Indolic GSL	*B. rapa*	Negative	Aghajanzadeh et al., 2015
O_3_	176 nL/L; 150 nL/L; 15 ∼ 20 ppb; 150 ppb; 60 ppb	Total GSLs	*B. napus*, *B. nigra*, *B. rapa*; *B. campestris*	Negative	Gielen et al., 2006; Himanen et al., 2008; Khaling et al., 2015; Han et al., 2020, 2021
	150 ppb; 60 ppb	Aromatic GSL	*B. campestris*	Negative	Han et al., 2020, 2021
CO_2_	600 ppm; 700 ∼ 1,000 ppm	Total GSLs; glucoraphanin and sulforaphane	*B. oleracea* L. var. *italica*; *B. oleracea* var. *alboglabra*	Positive	Almuhayawi et al., 2020; Chowdhury et al., 2021
**Chemical pollution**					
Imidacloprid, pyramezone, beta-cypermethrin and acephate	0.7 g/L; 0.5 g/L; 0.6 g/L; 2 g/L	Total GSLs and individual GSL	*B. rapa*	Positive	Zhu et al., 2015
Fluridon	0.5 μM	Total GSLs	*B. oleracea* L. var. *capitata* L.	Negative	Wang et al., 2015
AMP; GABA	40 μM; 10 μM	Total GSLs	*B. rapa*	Positive	Thiruvengadam et al., 2016b
Putrescine	100 μM	4-hydroxyglucobrassicin and gluconasturtiin	*B. rapa*	Positive	Thiruvengadam and Chung, 2015
Carbenicillin; penicillin	100 mg/L	Indole GSL	*A. thaliana*	Negative	Gudio et al., 2018

**FIGURE 3:**
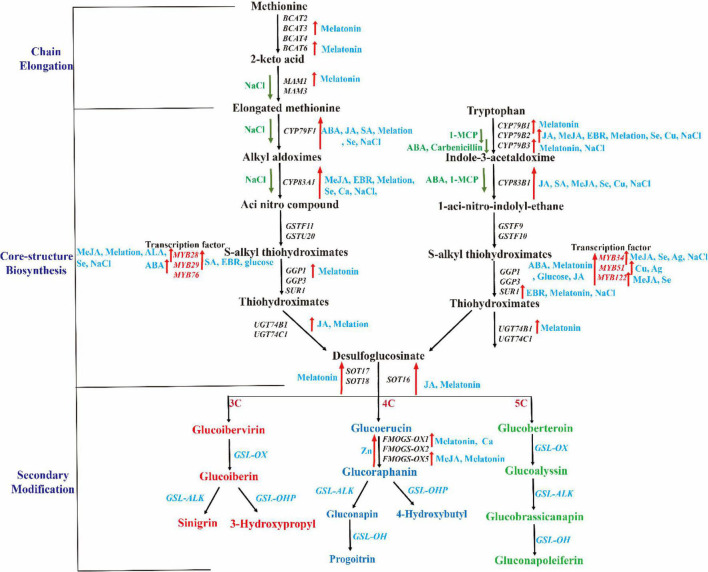
Regulation of different exogenous substances in Cruciferous crops GSL synthesis. The black italics and red texts represent related biosynthetic genes and transcription factors, respectively. The red and green arrows represent the gene expression increased or decreased after treatment with related chemicals.

With the development of molecular biology technology, the molecular biological mechanism of GSL biosynthesis, accumulation, and transport have made great progress recently, especially in *A. thaliana* (Barco and Clay, 2019; Kim et al., 2020). A large number of studies have shown that GSL biosynthesis is a complex process, which is regulated by multiple layers comprising genetic sites, developmental processes, transcription, and synthetic products (Barco and Clay, 2019; Meier et al., 2019). At present, the biosynthetic framework roadmap of GSL is basically clear in *A. thaliana*, and most regulatory genes in related pathways are known, but the specific functions of these regulatory genes are not fully elucidated (Ashari et al., 2018; Vo et al., 2018). In this process, *MYB* transcription factors play an important role in the regulation of GSL formation. The regulation pathway can be summarized as: *MYB* transcription factors are stimulated; *MYB* further induces or inhibits the expression levels of *BCAT4*, *MAM1*, *MAM3*, and other key genes of GSL biosynthesis in response to exogenous stimuli, thus achieving positive or negative regulation of GSL biosynthesis (Peskan-Berghofer et al., 2015; Zhou and Memelink, 2016; Harus et al., 2020). Therefore, a study of the regulation of *MYB* transcription factors and other GSL biosynthesis genes could provide a theoretical basis for molecular breeding and high-quality cultivation of cruciferous vegetable crops. Different from *A. thaliana*, the biosynthesis and regulation *MYB* genes of GSL in most cruciferous crops have multiple copies (Yang et al., 2021); there may be differences in the function of the same genes among different species of plants and resulting in much more complex studies. Through this gene redundancy, vegetable crops can rapidly induce the synthesis of GSLs when subjected to stress and improve their defense ability through high content and multi-component GSLs (Yang et al., 2021). However, this also leads to a more complex regulatory network for GSL biosynthesis in these crops, requiring a lot of work to explore their regulatory mechanisms in terms of upstream and downstream transcription factors. Besides, it is also not clear whether transcription factors, combined with the downstream target genes encoding interaction proteins, work together, nor whether, after the addition or diminution of exogenous chemicals, the resultant high content of GSLs is involved in feedback to regulate the biosynthesis of GSLs, which require further a in-depth study to reveal the complex GSL biosynthetic regulation network (Figure 3). Through the study of GSL biosynthesis and regulatory networks, we could elucidate the exogenous stimulus response mechanism of transcription factors and the mechanism regulating GSL synthesis induction, further enriching our knowledge of the regulation network of GSL synthesis, and providing a theoretical basis for molecular breeding, high-quality cultivation, and biological control of diseases and insect pests of cruciferous vegetables with high GSL contents.

The well-researched GSL biosynthesis regulation genes provide a direction for future Cruciferae molecular breeding. It is well-known that traditional breeding is time-consuming and difficult to change plant traits. Through molecular breeding, we can improve the content of beneficial GSL and reduce the content of harmful GSL in Cruciferae. For example, in the aliphatic GSL biosynthesis pathway, the anticancer glucoraphanin is first synthesized, and then the downstream 3-butenyl GSL is biosynthesized using glucoraphanin as raw material, which is then used to biosynthesize the harmful DL-GOITRIN (2-hydroxy-3-butenyl GSL). Through genetic engineering (e.g., RNA interference technology and gene editing technology), by knocking out the related genes that were involved in the subsequent biosynthesis after glucoraphanin, we can stop the process at the biosynthesis stage of sulforaphanin or making a very small amount of downstream GSL. In addition, because the biosynthesis of GSL has tissue specificity, the GSL can be transported after biosynthesis, so harmful GSL can be transported to non-edible organs and beneficial GSL can be transported to edible organs. By this method, high beneficial GSL plant products can be obtained while improving plant resistance to pests and diseases. Although there are a few reports on this aspect, the effect is not obvious and needs further research, especially the new gene editing technology has not been widely used in this research area (Abuyusuf et al., 2018; Petersen et al., 2019; Neequaye et al., 2021; He et al., 2022). It is believed that, in the near future, with the development of omics and gene editing technology, there will be a new understanding of the function and interaction network of GSL biosynthesis genes. Moreover, we can regulate GSL content and composition by functional editing of GSLs biosynthesis genes as needed.

## Author Contributions

ZL, HW, and JY designed the research. JL, SL, JW, and LL prepared the manuscript. LH, GZ, and JX revised the manuscript. All authors have read and agreed to the published version of the manuscript.

## Conflict of Interest

The authors declare that the research was conducted in the absence of any commercial or financial relationships that could be construed as a potential conflict of interest.

## Publisher’s Note

All claims expressed in this article are solely those of the authors and do not necessarily represent those of their affiliated organizations, or those of the publisher, the editors and the reviewers. Any product that may be evaluated in this article, or claim that may be made by its manufacturer, is not guaranteed or endorsed by the publisher.
